# In-Tube Solid Phase Microextraction: Basic Concepts and Recent Applications in Food Matrices

**DOI:** 10.3390/molecules31040730

**Published:** 2026-02-20

**Authors:** Maria Flávia Assunção Magalhães, Rafael Oliveira Martins, Josicleia Oliveira Costa, Jussara da Silva Alves, Fernando Mauro Lanças

**Affiliations:** Institute of Chemistry at São Carlos, University of São Paulo, São Carlos 13566-590, SP, Brazil; mfamag@usp.br (M.F.A.M.); rafaelom144@gmail.com (R.O.M.); cleia.95oliveira@gmail.com (J.O.C.); jussaraalves@usp.br (J.d.S.A.)

**Keywords:** In-tube solid phase microextraction, food analysis, miniaturized sample preparation, SPME optimization, sorbent materials

## Abstract

In-tube solid-phase microextraction (IT-SPME) is an advanced microextraction technique in which a sample solution flows through a capillary containing an internal stationary phase, enabling efficient extraction and preconcentration of target analytes. The online coupling to liquid chromatography is a key advantage of this technique, enabling full automation and high analytical throughput, both of which are significant for food analysis. Recent advances have focused on developing novel sorbent materials that respond to external stimuli (e.g., magnetic, electrical, or thermal) and on integrating them into emerging chromatographic platforms. Moreover, key operational parameters, including sample volume, pH, phase thickness, and the capillary’s dimensions (length and inner diameter), must be optimized to achieve enhanced selectivity, speed, and sensitivity. Despite this, the literature still lacks updated reviews of SPME concepts and their innovations for versatile applications in food matrices. Hence, this review outlines the fundamental principles of IT-SPME while highlighting key parameters that affect analytical performance. Finally, we provide a literature review of SPME applications in food analysis over the past 6 years, while exploring current trends and future directions for SPME development and enhanced applications in food science.

## 1. Introduction

With the advancement of green chemistry, the pursuit of more sustainable, simpler, faster, and more efficient methods has become a global priority in contemporary analytical developments. Since sample preparation is a critical step in the analysis of complex matrices, reducing the time and resources required at this stage is essential for developing more environmentally friendly methodologies [1]. In this context, techniques based on solid-phase microextraction (SPME) have gained increasing relevance [2,3,4,5]. In conventional SPME, a fiber coated with an extracting phase is inserted into the sample matrix to extract analytes under equilibrium conditions [6]. However, limitations such as fiber fragility and low sorption capacity prompted the development of in-tube solid phase microextraction (IT-SPME), initially proposed by Eisert and Pawliszyn in 1997 [7]. In addition to fiber-SPME, other microextraction techniques, including stir bar sorptive extraction (SBSE) and liquid-phase microextraction (LPME), have been widely applied in food analysis. These approaches can be limited by automation and control of extraction parameters, whereas IT-SPME offers distinct advantages by enabling precise control of key variables and straightforward integration with the analytical system [8,9,10,11,12,13].

IT-SPME employs capillary tubes that are either packed or internally coated with an extraction phase. Under dynamic conditions, the sample solution flows through the capillary, enabling effective preconcentration and extraction of the target analytes, which are subsequently desorbed by applying an appropriate solvent through the column [14]. One of the main advantages of this technique is the ability to integrate sample preparation and separation/detection steps online [15,16]. Such integration can shorten the overall analysis time, enhance the reliability of the procedure, and minimize solvent consumption. Investigations exploring the IT-SPME technique can be based on the following aspects: (1) Column dimensions and geometry, influencing analysis time and the feasibility of coupling with liquid chromatography (LC); (2) New extraction phases, improving extraction efficiency and selectivity; and (3) Coupling with liquid chromatography, including conventional LC, ultra performance liquid chromatography (UPLC), capillary liquid chromatography (CapLC), and nano liquid chromatography (NanoLC).

The column dimensions and geometry are directly related to analysis time and LC coupling feasibility, as the capillary i.d. must be optimized for the intended separation technique [17]. Various types of capillary columns are available, encompassing diverse synthesis and fabrication routes, from the selection of capillary material to the physicochemical properties of the final device. IT-SPME is a solid-based technique, and the extraction phase plays a crucial role not only in extraction efficiency but also in the device’s stability and robustness. Its performance depends on the target analytes and the sample matrix. Moreover, the ideal sorbent material must account for the packing device used in IT-SPME and ensure the system’s stability within the device. Coupling IT-SPME with LC systems enables online separation and detection of target analytes, thereby reducing analysis time and improving reproducibility and sensitivity for complex samples, particularly food matrices [18,19].

Although several reviews on IT-SPME have been published in recent years [15,16,20,21,22,23,24,25,26,27], these works primarily focus on fundamental principles, general device configurations, coating materials, and strategies applied across a wide range of matrices. In this context, the present work provides a comprehensive overview of IT-SPME applications in food sample preparation reported over the last six years, with particular emphasis on recent advances in coating materials, automated online configurations, and the specific challenges associated with complex food matrices, thereby addressing current analytical demands in food science.

## 2. Extraction Devices: Key Features and Configurations

IT-SPME devices exhibit architectural features critical to their performance in analyte extraction, particularly the morphology and chemical nature of the extraction phase, as well as the physical dimensions of the capillary tube. The geometry of the extraction phase can influence key operational parameters, including flow behavior, extraction capacity, mechanical stability, and compatibility with high-pressure chromatographic systems. Beyond the internal coating, the choice of capillary material directly affects the device’s mechanical strength and durability. Tube dimensions, such as internal diameter (i.d.), length, and dead volume, also play a central role in determining analysis time and extraction efficiency. Tubes with smaller internal diameters provide higher efficiency due to their higher surface-area-to-volume ratio, whereas longer columns can improve the technique’s sensitivity [28]. The availability of multiple IT-SPME configurations enables the technique to be tailored to specific analytical requirements, thereby reinforcing its applicability as a versatile and sustainable sample-preparation approach. Furthermore, this section discusses the principal geometries, materials, and dimensional parameters of capillaries used in IT-SPME.

### 2.1. Capillary Columns

In its classical configuration, IT-SPME employs an open-tubular capillary column as the extraction device. These columns are typically made of fused silica and coated with a stationary phase. In practice, the analytes in the sample are extracted and preconcentrated onto the stationary phase via adsorption or absorption. Only the fraction required for analytical detection is subsequently transferred [7]. Over the years, various capillary geometries and materials have been developed to expand the scope of the technique. Currently, various capillary types and extraction phases are available in IT-SPME. This enables the extraction of multiple analyte classes from diverse matrices [7,13]. Capillary columns are generally classified into four categories based on the geometry of the extraction phase: internally coated, particle-packed, fiber-packed, and monolithic columns, each providing distinct performance characteristics [13]. While the specifics differ across types, all benefit from tailored designs that optimize efficiency, stability, and selectivity, providing varying degrees of versatility across applications.

**Figure 1:**
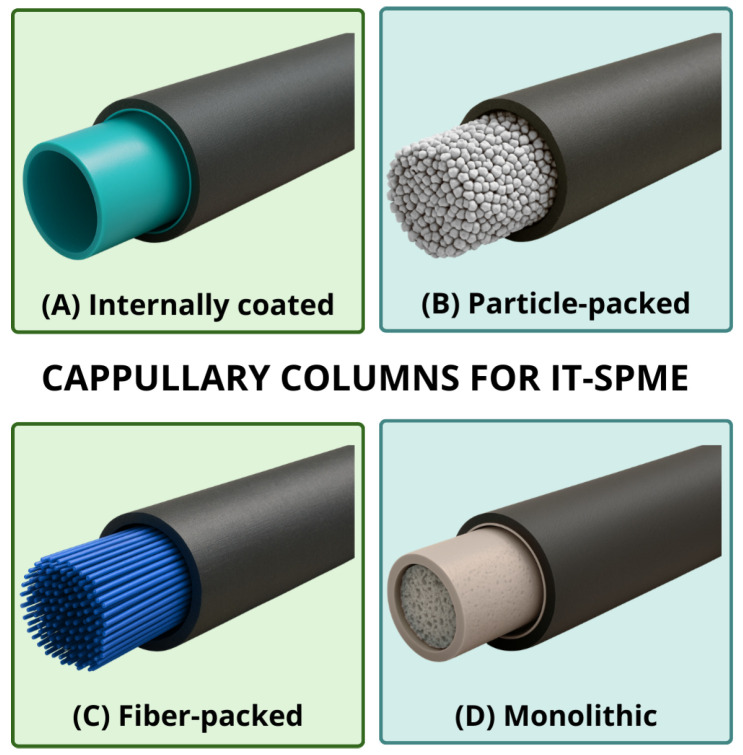
Schematic illustration of different capillary column geometries used in IT-SPME: (**A**) internally coated; (**B**) particle-packed; (**C**) fiber-packed; and (**D**) monolithic column.

Internally coated capillaries (Figure 1A) consist of hollow tubes with inner walls covered by an adsorbent. They usually are classified as wall-coated open tubular (WCOT) or porous-layer open tubular (PLOT) columns [29]. Due to their low mobile-phase flow rate, these columns are well-suited to high-sensitivity applications, thereby reducing solvent consumption and waste generation [30]. In WCOT columns, the adsorbent is firmly bonded and crosslinked to the inner wall of the capillary, ensuring stability during solvent flow and preventing phase loss. Although the film is thin and reduces extraction capacity, these columns are particularly suitable for the analysis of small molecules [29]. Examples include TRB (35% diphenyl-65% polydimethylsiloxane) and DB-Wax (polyethylene glycol) columns [31]. PLOT columns operate via adsorption and feature a thick film (~17 μm) with a large surface area. This yields higher extraction efficiency but also makes them more susceptible to peeling and degradation, depending on the solvent. Examples include Supel Q PLOT and Carboxen 1006 PLOT column [29,31]. These columns have been used for the extraction of various compounds from food matrices, including flavones from herbal teas [32], ochratoxins from nuts and grains [33], and polycyclic aromatic hydrocarbons (PAHs) from honey [34].

Various commercially available columns originally developed for gas chromatography (GC) have been employed as extractors in IT-SPME. They use carbon-based sorbents (divinylbenzene polymers, Carboxen, and carbon molecular sieves) or silica-based sorbents (polydimethylsiloxane (PDMS), diphenyl-PDMS, and cyanopropylphenylmethylpolysiloxane). These columns offer reliable performance and can be reused multiple times. Their versatility enables efficient extraction of compounds with differing polarity and porosity [29]. Extraction efficiency depends on the analyte’s affinity for the capillary phase, making the choice of sorbent critical. To enhance selectivity and overall performance, functional phases have been developed, including carbon-based nanomaterials, such as carbon nanotubes (CNTs), graphene, monoliths, restricted-access materials (RAM), molecularly imprinted polymers (MIPs), ionic liquids (ILs), metal–organic frameworks (MOFs), and deep eutectic solvents (DESs) [29]. Mixtures of polymers and nanomaterials can be immobilized on the capillary wall, enhancing selectivity and durability [29,30]. These sorbent materials, commonly referred to as laboratory-made phases, have been introduced not only to enhance extraction performance for target analytes in complex samples but also to mitigate the considerable costs associated with commercial IT-SPME columns, which often require substantial resources for purchase.

Besides internally coated capillaries, another widely studied geometry is packed-particle columns (Figure 1B), in which adsorbent-coated particles are packed into the tube [29]. Analogous to solid-phase extraction (SPE), these particles or microspheres, possessing sorptive properties, optimize space utilization and enhance extraction performance [24]. Packed particles can be further enhanced by incorporating advanced sorbent materials. Carbon-based nanomaterials, such as graphene and graphene oxide (GO), have been explored in IT-SPME owing to their high surface area and diverse mechanisms of analyte interaction, including polar and hydrophobic interactions. Silica-functionalized derivatives have also demonstrated good efficiency, particularly for specific compounds such as xanthines [24].

Appropriate material selection enhances the overall performance of packed columns. However, this geometry exhibits intrinsic characteristics affecting extraction efficiency. In general, packed particle columns offer advantages such as higher surface area and retention capacity. Still, they present limitations, including clogging, susceptibility to high pressures, and the need for careful preparation of samples and extraction devices. Sorbent particles should possess a high surface area, a suitable pore structure, functional groups, and resistance to interferences, ensuring reliability and robustness [24].

Building upon the concept of packed particle columns, another relevant geometry is packed fiber capillary columns (Figure 1C), which combine features of fiber SPME and IT-SPME [23]. In this configuration, thin fibers are packed vertically within the tube, resulting in higher extraction efficiency and a lower pressure drop than internally coated capillary columns [25,35,36]. The fibers are arranged in parallel, forming channels that optimize sample flow. Compared with monolithic capillary columns and on-line SPE, packed fibers offer a lower risk of clogging, good reusability, and comparable efficiency and resolution [36].

Fibers can be used in their bare form, though retention capacity varies by analyte class. To overcome these limitations, innovative coatings, such as metallic fibers, functionalized polymers, and composites, have been developed, thereby enhancing the durability, selectivity, and extraction efficiency of the devices [36]. Among these advances, the use of metallic supports instead of silica stands out, increasing the fibers’ lifespan and mechanical robustness [37]. A recent example is the application of packed fibers made of covalently functionalized carbon (CFs@C-COF) in IT-SPME for the extraction of phthalates from Baijiu, demonstrating high extraction efficiency and sorbent durability [38]. The fiber packing density (interstitial fraction, ε_ᵢ_) is a critical parameter that influences column permeability, mass transport, and extraction efficiency. Extreme densities compromise the balance between permeability and binding capacity, making the optimization of ε_ᵢ_ essential [39].

Beyond particle- and fiber-packed columns, monoliths offer another essential approach for IT-SPME (Figure 1D). These columns consist of a continuous monolithic structure with a bimodal porous network, in which macropores provide permeability and low pressure drop. At the same time, mesopores increase surface area and retention capacity, resulting in a greater number of active sites than in internally coated columns [31]. Compared to packed columns, monoliths do not require frits and can be prepared via in situ polymerization. Such a strategy provides greater control over porosity and extraction efficiency in the final application [31,40].

Inorganic silica monoliths are prepared via the sol–gel process, involving the hydrolysis and condensation of precursors in the presence of a catalyst and a porogenic agent. Their bimodal structure provides high permeability, large surface area, and efficient extraction of small analytes. However, they exhibit low tolerance to pH variations, require functionalization, may undergo shrinkage, and possess limited biocompatibility [40]. In addition to inorganic monoliths, organic polymers represent a versatile alternative, particularly for applications involving biological samples. Polymeric monoliths obtained by radical polymerization in the presence of porogenic solvents exhibit high permeability, mechanical flexibility, and biocompatibility, making them suitable for use in biological matrices. However, they are less chemically stable and prone to shrinkage or swelling in organic solvents [31,40].

Commonly prepared from monomers such as styrene and acrylates, polymeric monoliths possess a monomodal macropore distribution suitable for high-molecular-weight compounds, including peptides and proteins. They maintain good loading capacity and surface area [15]. While inorganic monoliths provide high extraction efficiency for small analytes, polymeric monoliths excel at extracting larger biomolecules due to their porosity and biocompatibility [31,40]. These characteristics render polymeric monoliths effective and adaptable for extracting analytes across a broad molecular weight range in complex biological matrices.

For monolithic columns, incorporating functional nanomaterials, such as immobilized antibodies, further increases surface area and the number of active sites, thereby improving selectivity and extraction efficiency for biomolecules. Beyond pure monoliths, hybrid monoliths have emerged as an alternative by combining features of inorganic and polymeric monoliths, thereby providing increased stability, permeability, and enhanced selectivity [31]. Hybrid monoliths have been synthesized in situ within columns. Additionally, incorporating hybrid sorbents, such as immunosorbents and magnetic composites, further enhances versatility and enables selective extraction from complex samples [9]. Advances in functionalization, including the introduction of organic functional groups and mesoporous particles, have enabled greater morphological control and sorbent stability without compromising mass-transfer performance [31,40]. Hybrid monoliths integrating polymeric matrices with chemically bonded halloysite nanotubes (HNTs) have shown prolonged sorbent durability and enhanced adsorption capacity. They have demonstrated outstanding extraction efficiency for polar cationic pesticides in complex sample matrices such as fruits and vegetables [41].

Surface modification plays a crucial role in improving extraction efficiency and selectivity across IT-SPME configurations. Functionalization strategies, including the incorporation of nanoparticles, nanotubes, or polymeric blends, can enhance the sorbent’s surface area, stability, and affinity toward target analytes. These effects are consistent across different geometries, from coated capillaries to monolithic and hybrid formats, although the magnitude of improvement varies depending on the material and synthesis method.

### 2.2. Capillary Material and Dimensions

**Figure 2:**
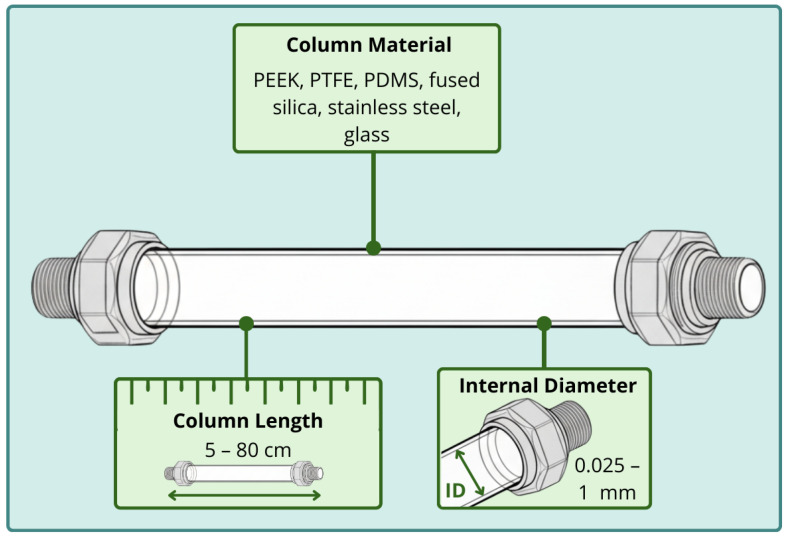
Schematic illustration of capillary materials and dimensions for IT-SPME columns.

As illustrated in Figure 2, the extraction performance can be influenced by the capillary material and column dimensions, such as length and internal diameter. The capillary material governs durability, chemical compatibility, and extraction efficiency in IT-SPME. Various materials can be employed, including fused silica, polyetheretherketone (PEEK), polytetrafluoroethylene (PTFE), PDMS, stainless steel, and glass, selected according to the application and desired properties. Fused silica capillaries typically have internal diameters (i.d.) ≤ 1.000 μm and polyimide outer coatings. They allow efficient phase immobilization via polymerization, chemical modification, or electrodeposition. However, ionized silanol groups on the inner wall can lead to nonspecific adsorption of biomolecules, reducing extraction efficiency [15].

Treated PEEK tubing supports direct polymerization of organic monoliths, providing flexibility and high-pressure resistance. This contrasts with the more fragile silica [15,30,31]. PTFE capillaries offer chemical stability and flexibility, while stainless steel provides durability and resistance to pressure and corrosion, making them particularly suitable for electrodeposition [15,31,41]. Plastic and stainless-steel capillaries used in online coupling have chemically inert surfaces that limit sorbent immobilization and are therefore more suitable for particle- or fiber-packed configurations [15]. Achieving stable and reproducible capillaries requires control over nonspecific adsorption, porosity, and extraction efficiency. For PEEK and PTFE, surface modifications, such as controlled etching, polydopamine coating, or incorporation of functional materials (graphene, chitosan, dialdehyde starch), enable overcoming chemical resistance, anchoring sorbents, and optimizing the extraction of both polar and nonpolar analytes [31]. PEEK, PTFE, and stainless steel capillaries are suitable for the analysis of protein-rich samples, such as meat products, as they tend to reduce the nonspecific adsorption of biomacromolecules when compared to fused silica capillaries.

Stainless steel is frequently employed as a support for electrodeposition, serving as the working electrode. Additionally, biocompatible substrates, such as nanostructured polypyrrole (PPy), have been explored to form porous networks that enable rapid extraction and direct coupling to mass spectrometry (MS) systems [31]. The selection of capillary material, combined with appropriate surface modification techniques, enables optimization of extraction performance and compatibility with a range of analytes. This also enhances the efficiency, stability, and versatility of the extraction devices and facilitates coupling with analytical systems, such as LC and MS [15].

Column length is critical for analyte recovery and chromatographic performance. Capillaries with lengths of 15–80 cm balance extraction efficiency and analysis time; 60–80 cm are most used in optimized setups [15,29]. The geometric dimensions of the capillary are critical to performance, particularly the inner diameter (i.d.) and coating thickness. Diameters ranging from 25 to 800 μm i.d. have been tested, with 320 μm i.d. being the most common, as it provides a balance between flow rate, pressure, and number of theoretical plates [15,31]. Shorter capillaries or larger internal diameters are often preferred for viscous or lipid-rich food matrices, such as milk and oils, as they reduce backpressure and minimize clogging risks.

In fused-silica capillaries, increasing the inner diameter (i.d.) tends to make them more fragile, requiring careful handling and installation [30]. A larger diameter increases extraction capacity but reduces the number of theoretical plates. It also increases dead volume, resulting in broader peaks and lower efficiency. For IT-SPME capillaries of varying i.d., selection should consider the coupling mode and mass-transfer requirements. The coating thickness is another critical parameter. Thin films (<1 μm) reach equilibrium faster but have limited capacity, whereas thicker coatings allow higher extraction efficiency but hinder desorption and exhibit lower mechanical stability. In monolithic or chemically coated columns, the firm anchoring of the sorbent phase to the inner wall mitigates these drawbacks, ensuring stability and reproducibility [15,40].

Short monolithic capillaries (5–10 cm; 200–530 μm i.d.) are commonly used in off-line or on-line LC modes—columns with i.d. <200 μm are preferred for CapLC or electrochromatography [15]. The selection of dimensions should balance extraction capacity, analysis time, phase stability, and compatibility with the analytical system. Overall, the choice of capillary geometry, material, and dimensions should be carefully optimized according to the target analytes and analytical application. Together, these parameters govern extraction performance, stability, and versatility, ultimately determining the sensitivity, reproducibility, and overall efficiency of IT-SPME systems.

The dimensions, capillary material, and phase geometry of IT-SPME columns define the physical framework of the technique; however, analytical performance is primarily governed by the extraction phase employed. Therefore, a detailed discussion of the materials used in IT-SPME is essential for understanding selectivity, extraction efficiency, and matrix tolerance in food analysis.

## 3. Sorbent Materials for IT-SPME: Advantages and Limitations Towards Current Strategies

As previously discussed, sorbent choice is one of the most important parameters governing the extraction performance of IT-SPME. Since the stationary phase mediates interactions with target compounds, selecting an appropriate coating determines the prevailing mechanisms of interaction, including hydrogen bonding, polar and dipole–dipole interactions, π-π stacking, and specific or nonspecific adsorption [15,23]. Therefore, coating selection should be guided by the physicochemical properties of the analytes and by the sorbent’s chemical composition, surface functionality, and morphology to maximize selectivity, capacity, and mass transfer efficiency. Different commercial GC stationary phases are available and are often grouped into carbon-based sorbents (e.g., cross-linked divinylbenzene polymers) and polysiloxane-based materials (e.g., polydimethylsiloxane and diphenyl-modified polydimethylsiloxane) [42]. These phases are widely used to manufacture WCOT and PLOT columns for diverse applications [20].

Despite the widespread use of conventional and commercial coatings for producing IT-SPME columns, ongoing advances in sample preparation have introduced numerous alternative materials that have proven highly effective as IT-SPME coatings. These materials have opened new possibilities for more selective phases while also addressing key physicochemical concerns, including mechanical and thermal stability, increased surface area, and, in many cases, a greener environmental profile compared to traditional materials [43]. In food analysis, the introduction of novel materials has provided notable advantages by enabling both sample cleanup and preconcentration of target analytes, thereby enabling diverse applications, such as contaminant monitoring and chemical profile evaluation of these matrices [37,43].

**Figure 3:**
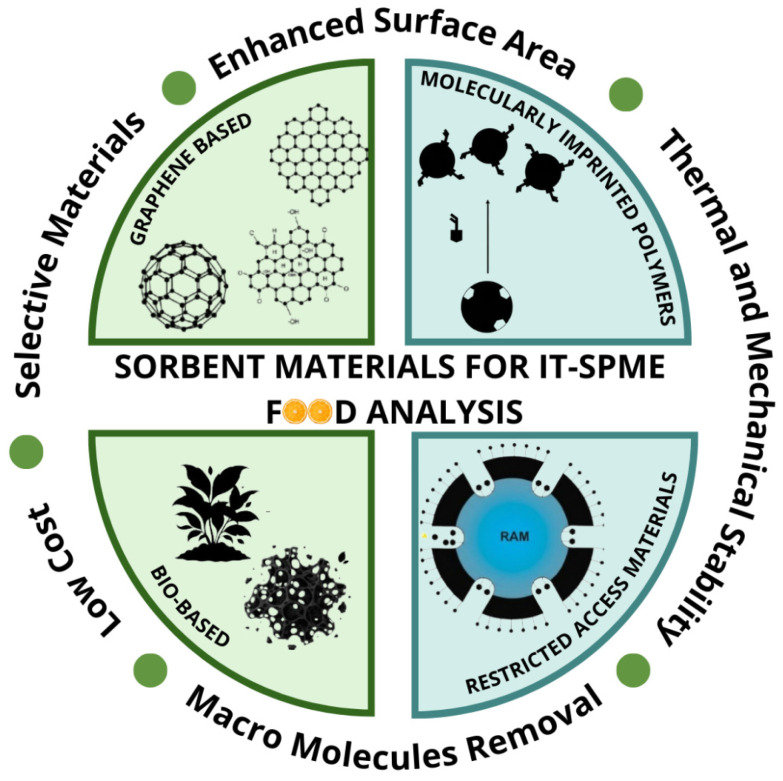
Schematic illustration of different sorbent materials for IT-SPME applications.

As schematically illustrated (Figure 3), distinct sorbent materials exhibit diverse characteristics: graphene-based materials have enhanced surface area and excellent thermal and mechanical stability, enabling high extraction efficiency. MIPs are highly selective materials and are particularly advantageous for the determination of specific contaminants. Bio-based materials are sustainable and low-cost alternatives, whereas RAMs enable the selective removal of macromolecules, such as proteins and lipids. Accordingly, this section offers a concise overview of coatings and alternative materials used to fabricate IT-SPME columns, with an emphasis on applications in food matrices.

### 3.1. Carbon-Nanomaterials

Since the introduction of graphene (G) in sample preparation, its outstanding physicochemical properties, such as its large surface area and ease of chemical modification, have made it a promising platform for developing novel phases [44,45]. Graphene derivatives, including GO and reduced graphene oxide (rGO), have been widely explored in various microextraction techniques and are increasingly applied to IT-SPME [46]. In addition to their high adsorption capacity, graphene-based materials offer remarkable versatility for creating hybrid coatings with improved selectivity, mechanical strength, and thermal stability. This versatility has opened new perspectives for IT-SPME applications [47].

**Figure 4:**
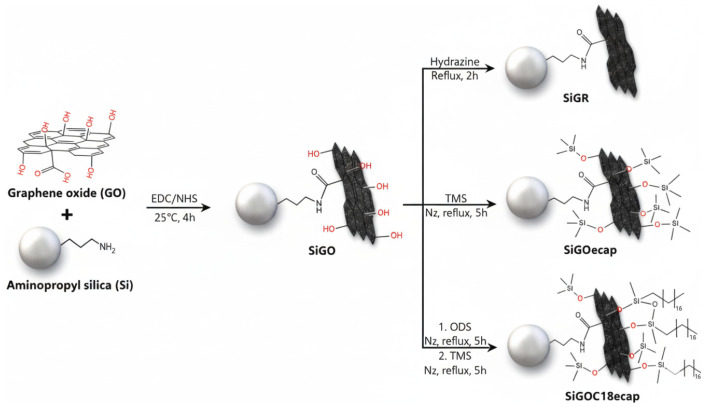
Schematic illustration of the synthesis protocol for obtaining SiGOC18ecap. Adapted from Carmona and Lanças [49].

GO has been used to fabricate a variety of IT-SPME columns. It is commonly prepared by the Hummers method, in which graphite is oxidized with concentrated sulfuric acid and potassium permanganate, then sonicated, thoroughly washed with deionized water, and dried to obtain the GO powder [48]. Carmona and Lanças [49] reported an aminopropyl silica support coated with graphene oxide and further functionalized with end-capped octadecylsilane (SiGOC18ecap) for IT-SPME extraction of xanthines in coffee beverages (Figure 4). The resulting coating enabled hydrophobic and π-π interactions with xanthines. In contrast, the silica anchoring enabled the use of a GO-based phase in a packed IT-SPME format, mitigating backpressure and clogging issues typically observed when GO particles are applied in packed devices. In another application, a GO-functionalized mesoporous silica sorbent was packed into a stainless-steel wire 32 cm long and 0.18 mm in diameter, and used to extract PAHs from honey [50]. A key advantage of the silica-graphene oxide (SiGO) sorbent was its durability, sustaining more than 110 consecutive runs without compromising analytical performance in this complex food matrix.

CNTs emerged in the early 1990s, when authors reported the production of multi-walled carbon nanotubes (MWNTs) by arc evaporation [51]. Structurally, CNTs are seamless cylinders of sp^2^-bonded carbon atoms arranged in a hexagonal or pentagonal shape. These structures are commonly divided into single-walled nanotubes (SWNTs), consisting of a single graphene cylinder with diameters typically 0.7 to 2 nm, and MWNTs, comprising concentric graphene cylinders with outer diameters ranging from a few to several tens of nanometers [52,53]. Numerous synthesis routes have been described, including arc evaporation, laser ablation, and catalytic chemical vapor deposition, each offering different trade-offs in yield, diameter control, alignment, and purity [54]. CNTs combine high tensile strength, excellent electrical and thermal conductivity, and very low density with a large specific surface area and rich π-electron surfaces, properties that make them attractive sorbent materials for sample preparation, including IT-SPME methods. Loussala et al. [55] proposed silica functionalized CNTs for IT-SPME extraction of PAHs from bottled water. The IT-SPME device was prepared by coating a 32 cm-long, 0.18 mm-diameter stainless-steel wire with epoxy resin, then adhering silica-functionalized CNTs to the epoxy-coated surface, and drying for 36 h. Morphological characterization showed granular structures with abundant cavities and interparticle pores, which enhanced mass transfer and provided numerous adsorption sites for interaction with the target analytes.

Despite the remarkable advantages of graphene-based coatings and the breadth of materials they offer, most IT-SPME applications in food have concentrated on SiGO packed into stainless-steel wires. Even so, their use in food matrices remains less common than in biological or environmental analyses. This underrepresentation likely reflects challenges intrinsic to carbon-based materials in IT-SPME, including limitations stemming from their physicochemical properties and practical constraints within the microextraction workflow.

1. **Pressure and clogging:** Agglomeration of sheets and migration of fines inside the capillary can generate high backpressure, destabilize pump loads, cause leaks, and even block the column.
2. **Poor packing reproducibility and channeling:** Because particle size, density, and surface chemistry are hard to control, forming a uniform bed is difficult. Resulting voids promote channeling, lower extraction efficiency, and distort peak shapes when the device is coupled to LC.
3. **Chemical variability between batches:** When synthesis is not standardized, the resulting carbon materials differ in their physicochemical properties. In IT-SPME, this leads to inconsistent capacity, selectivity, and overall analytical performance during method development.
4. **Mechanical fragility of coatings:** Carbon-based coatings can delaminate or fracture under pressure and flow, releasing fines, increasing backpressure, and shortening the device’s usable lifetime.

The use of carbon-based materials as coating sorbents in IT-SPME offers clear advantages, notably their high surface area and rich interaction mechanisms. Nevertheless, the literature shows that their application in IT-SPME for food analysis remains limited. Targeted studies are still needed to elucidate their potential fully and to guide the design of tailored approaches for complex food matrices. In particular, carbon nanofibers (CNFs), fullerenes, and other carbon allotropes are particularly promising for IT-SPME in food science but remain largely underexplored.

### 3.2. Molecularly Imprinted Polymers (MIPs)

Another important class of materials explored in sample preparation is MIPs. MIPs are known primarily for their ability to selectively recognize target compounds, making them a strong choice when specific binding is required in complex matrices [56]. Their selectivity arises from their synthesis protocol, which works as a key-locker mechanism. During polymerization, a template molecule is combined with functional monomers and crosslinkers in the reaction medium. The template molecule is typically represented by the analyte of interest or a close structural analog [57]. As the polymer forms, cavities complementary to the template in size, shape, and functional group orientation are created within the three-dimensional network. After polymerization, washing steps remove the template using an appropriate solvent, leaving recognition sites that can selectively bind analytes that share the template’s key features [56,58].

A persistent challenge is the complete removal of the template. A residual template trapped in the polymer can leach during extraction, leading to false positives or overestimated recoveries. To mitigate this, many protocols employ a dummy template; that is, a compound that closely mimics the target analyte’s relevant properties but is analytically distinguishable. Using a dummy template reduces the risk of template bleeding while preserving the specificity of the imprinted cavities [59,60]. Another concern in the use of MIPs for sample preparation is their structural fragility. Inefficient template removal can disrupt the three-dimensional polymer network and introduce defects. Although MIPs often show broad stability under harsh extraction conditions, prolonged or repeated exposure to extreme pH, oxidants, organic solvents, or high temperatures can still damage the polymer, shorten its service life, and degrade analytical performance [61]. Despite the challenges, MIPs remain valuable materials with unlimited potential for application in IT-SPME for food matrices.

Souza et al. [62] employed MIPs as a coating sorbent for IT-SPME to extract parabens from breast milk. The IT-SPME device used a fused-silica capillary (0.53 mm inner diameter, 5.0 cm length) that was activated with 1.0 mol L^−1^ NaOH solution, followed by 0.1 mol L^−1^ HCl solution. The MIP was synthesized with benzylparaben as the template, 4-vinylpyridine as the functional monomer, ethylene glycol dimethacrylate as the crosslinker, and 2,2-azobisisobutyronitrile as the initiator. Recognition assays showed that the MIP-packed IT-SPME provided superior extraction compared with the corresponding non-imprinted polymer (NIP), which was synthesized without the template. The specific binding cavities created by benzylparaben imprinting conferred higher selectivity and capacity than the nonspecific interactions of the NIP, enabling improved extraction in the complex breast-milk matrix.

In another food application, MIPs were employed to extract fluoroquinolones and sulfonamides from pork liver [63]. For the IT-SPME device, silica fibers 10.0 cm long and 125 μm in diameter were first modified and silanized. The MIP was synthesized using ofloxacin as the template, methacrylic acid as the functional monomer, and trimethylolpropane trimethacrylate as the crosslinker, with polymerization carried out at 60 °C. The MIP-coated silica fibers were then manually inserted into a hollow PEEK tube with 0.50 mm inner diameter and connected to a six-port valve in place of the injection loop. Selectivity was confirmed against NIP fibers, and enrichment factors from 69 to 136 demonstrated the effectiveness of the specific recognition cavities for antibiotics in the food matrix.

Despite the clear advantages of using MIPs in IT-SPME applications for food samples, the literature still lacks recent examples of their combination with this technique for food analysis. Moreover, despite the remarkable features of MIPs, critical concerns may limit their acceptance in IT-SPME applications, particularly relative to traditional, well-established packed materials. The challenges associated with their use can be summarized as follows.

1. **Template removal and residual contamination:** As previously mentioned, one of the main challenges in applying MIPs to IT-SPME is the inefficient removal of the template molecule from the polymeric network. Incomplete template elimination can lead to carry-over effects and false-positive interpretations in both qualitative and quantitative analyses.
2. **Heterogeneity of binding sites:** Controlling the final physicochemical properties of MIPs remains difficult, as non-standardized synthesis procedures often result in non-uniform binding sites. This heterogeneity affects the affinity toward target analytes and compromises the reproducibility and accuracy of the extraction process.
3. **Mechanical fragility and pressure buildup:** MIPs are often brittle, and during packing or operation under flow conditions, they may generate fine particles. These fines can cause high backpressure, channeling, and even capillary blockage during IT-SPME operation, thereby reducing system stability and analytical performance.
4. **Difficulties in packing and immobilization:** Achieving a uniform and stable packing within capillary columns is technically demanding. Irregular particle morphology and weak adhesion to the capillary wall can lead to bed instability, uneven flow distribution, and poor reproducibility during IT-SPME applications.

### 3.3. Restricted Access Material (RAM)

RAM is a family of sorbents designed for sample preparation with the specific purpose of excluding macromolecules while retaining low-molecular-weight analytes. Their working principle combines a diffusion barrier that prevents large species such as proteins from reaching the active sites with physicochemical interactions that capture small targets, including partitioning, ion exchange, hydrophobic interactions, and adsorption [64,65]. By blocking these macromolecules at the outer surface and allowing only small molecules to diffuse into the sorbent, these materials reduce fouling, minimize matrix effects, and improve selectivity [66]. They are particularly valuable for protein-rich samples commonly used in food analysis, such as meat and milk, where they yield cleaner extracts and more reliable quantification.

The literature describes various synthesis strategies for obtaining RAMs. These materials are commonly classified into four main categories, each with distinct synthesis approaches, advantages, and limitations: physical unimodal phases, physical bimodal phases, chemical unimodal phases, and chemical bimodal phases. Although each category has unique features, they share the general benefits of RAMs, including the ability to exclude macromolecules, excellent reusability, and compatibility with LC methods coupled to sample preparation strategies, among others. RAMs have found a special place in IT-SPME applications, demonstrating their potential across various uses; however, there are still few studies on their use in food monitoring [67].

A notable example of its potential in IT-SPME was reported by Souza et al. [61] for the determination of parabens in human milk. In that study, a hydrophilic outer layer was created during MIP formation inside the IT-SPME capillary. This external hydrophilic layer acted as a chemical diffusion barrier, forming a non-adsorptive network that excluded macromolecules from the milk matrix and facilitated the efficient binding of the target parabens to the MIP. The bibliographic review in this study clearly showed that RAMs in IT-SPME have been predominantly applied to biological analyses [68,69]. This was the first application of RAM in IT-SPME [70,71], highlighting the still untapped potential for further exploration of this material–technique combination in food research.

Interestingly, a RAM-based approach closely related to IT-SPME has been reported for the analysis of mebendazole and albendazole in milk using online SPE [72]. The method employed a restricted-access molecularly imprinted polymer (RAMIP) packed into a column and connected via an electronic six-port valve. Although that study did not use IT-SPME, the methodological similarity encourages researchers to invest in online strategies and supports the further adoption of RAMs in IT-SPME for food applications.

As previously discussed, RAM sorbents present several challenges and drawbacks associated with their specific physicochemical properties and their use in IT-SPME. The key aspects are summarized below:

1. **Surface fouling and clogging:** Despite the exclusion mechanism, proteins and other macromolecules may still adsorb non-specifically on the outer layer, gradually blocking pores and increasing system backpressure.
2. **Instability under extreme conditions:** RAMs often show limited stability at extreme pH values. In addition, high temperatures and strong organic solvents used in some extraction protocols can damage the material and compromise its integrity.
3. **Limited retention for small analytes:** Depending on the synthesis route and final architecture, the RAM phase may provide insufficient retention for small molecules. This limitation is critical with large sample volumes or multi-analyte extractions.
4. **Flow and pressure instability in IT-SPME capillaries:** Pore size distribution, particle packing heterogeneity, and related factors can lead to elevated and unstable backpressure in the IT-SPME format, which may strain pumps and valves and risk instrument damage.
5. **Batch-to-batch variability:** RAM synthesis is typically complex and multistep. Variations between batches in layer thickness, porosity, and surface chemistry can affect reproducibility and other critical method-performance metrics.
6. **Connector and dead-volume effects:** In IT-SPME, interfaces between the RAM bed and tubing can create mixing zones that dilute sample plugs, leading to peak broadening and poorer peak shape.

### 3.4. Bio-Based Materials

The term bio-based materials refers to those obtained through natural, non-aggressive environmental routes [47]. They include biopolymers derived from natural sources, such as bacteria, fungi, plants, and certain animals. Typical examples include chitosan, cellulose, and cyclodextrin. Natural materials derived from agricultural or industrial waste have also been used in solid-based sample-preparation methods [73]. Among their main advantages, the abundance of functional organic groups in their inherent structures is a key factor that underpins their versatility in producing hybrid materials for various sample-preparation methods, including IT-SPME for food samples [74].

A chitosan–polyvinyl alcohol electrospun nanofiber was applied as an IT-SPME coating to evaluate acidic red dyes in juice samples [75]. For fiber fabrication, the authors prepared a polyvinyl alcohol solution and a 2 wt% chitosan solution in acetic acid, then mixed them at a 20:80 chitosan-to-polyvinyl alcohol ratio. The mixture was stirred for 1 h at 80 °C, then electrospun using a high voltage power supply and a homemade collector. For the IT-SPME, a stainless-steel tube measuring 50 mm in length, 2.97 mm in outer diameter, and 1.80 mm in inner diameter, together with nine stainless-steel wires, was used. Chemical characterization showed that the biofibers had a smaller average diameter (212.71 nm) than fibers made from polyvinyl alcohol alone. It exhibited a rich profile of NH_2_ groups, facilitating the extraction of acidic red dyes from food matrices.

Food applications of bio-based materials as coating phases for IT-SPME are still largely unexplored, as most studies in the literature have focused on their use for biological and environmental sample analysis [76,77,78]. Several challenges still hinder their application in sample-preparation methods, including the development of IT-SPME. These limitations can be summarized as follows.

1. **Chemical stability:** Many bio-based biopolymers are sensitive to extreme extraction conditions, including pH, temperature, ionic strength, and common organic solvents. This instability can alter their properties and compromise extraction performance in IT-SPME.
2. **Mechanical robustness:** In electrospun films and other soft biopolymer layers, structural integrity may be compromised under compression inside capillaries, reducing lifetime and reproducibility.
3. **Packing reproducibility:** Controlling fiber diameter and packing homogeneity is challenging, which affects porosity and bed density and leads to variable backpressure between runs.
4. **Surface fouling:** Proteins, lipids, and polysaccharides from food matrices can irreversibly foul hydrophilic or charged surfaces, even when exclusion mechanisms are present.
5. **Functional-group control:** Achieving uniform, stable densities of amino, hydroxyl, or carboxyl groups from natural precursors is challenging and can affect selectivity and capacity.
6. **Batch-to-batch variability:** Variations in synthesis can change pore size, morphology, and other features, affecting performance. In addition, lab-prepared natural materials often require extensive purification to be suitable for use as IT-SPME coatings, thereby increasing the number of steps, resources, and time required.

### 3.5. Other Materials Applied to IT-SPME in Food Analysis

As the field of sample preparation has evolved, so too has the introduction of novel materials for solid-phase extraction protocols. These materials are not limited to those discussed in Section 3. In this section, we briefly explore additional promising materials and their applications in IT-SPME for food analysis.

ILs are salts composed of bulky organic ions that are liquid at or near room temperature. Typical cations include imidazolium, pyridinium, and pyrrolidinium, paired with a variety of anions. Compared with conventional sample-preparation materials, ILs are often considered greener and offer distinct physicochemical properties, including tunable viscosity, negligible vapor pressure, and high thermal and mechanical stability [79]. In sample preparation, their use has focused on developing novel sorbents and coating phases for microextraction techniques, such as IT-SPME. Tap water samples were analyzed by IT-SPME using carbon fibers coated with a melamine–formaldehyde aerogel modified with an ionic liquid for the extraction of estrogens [80]. The coating was prepared on 45 cm carbon fibers, and the ionic liquid 1-dodecyl-3-(3-aminopropyl)imidazolium bromide was synthesized from 1-bromododecane and 1-(3-aminopropyl)imidazole in a 1:1 molar ratio. Durability tests showed the fibers could be reused more than 150 times without loss of analytical performance. The application to tap water demonstrated effective extraction of estrogens.

Magnetic nanomaterials (MNMs) are notable for their superparamagnetic behavior and are widely used in magnetic solid phase extraction (MSPE). Their large surface area and tunable surface chemistry make them valuable in sample preparation [81]. Beyond MSPE, they have also been integrated into IT-SPME. Mei et al. [82] developed a magnetism-enhanced monolithic coating for IT-SPME to extract parabens from complex samples, including grape juice. In their setup, capillaries bearing the magnetic coating were connected to positions 1 and 4 of valve 1 in a six-port valve system. The applied magnetic field generated gradients that concentrated diamagnetic analytes into regions of minimal field within the monolith. During desorption, reversing the magnetic field facilitated analyte release, which improved overall analytical performance for parabens in food matrices.

Aerogels are highly porous materials produced by replacing the liquid in a wet gel with a gas while preserving the gel network. Their properties depend on the synthesis conditions, which control pore size, structure, and adsorption capacity [83]. In IT-SPME, a melamine formaldehyde aerogel functionalized with silver nanoparticles has been used to extract PAHs from drinking water [84]. The aerogel was prepared by condensing melamine with formaldehyde in the presence of sodium carbonate at 80 °C, followed by freeze drying to obtain the porous solid. It was then modified with silver nanoparticles and applied as the IT-SPME coating in a 30 cm length PEEK tube. Although aerogels show strong potential for IT-SPME, most applications to date have targeted environmental and drinking water samples [85,86]. While drinking water can be considered a food matrix for method development, studies on more complex food matrices remain scarce. Demonstrating robust performance in these matrices is essential to fully establish the importance of aerogel-based coatings for IT-SPME in the broader field of food analysis.

Monolithic capillaries are well established in IT-SPME. They consist of a single, continuous porous structure with interconnected macropores and mesopores. In IT-SPME, they are valued for their lower column backpressure, faster mass transfer, and higher extraction capacity than open-tubular columns. Monolithic formats include silica-based monoliths, organic polymer monoliths, and hybrids that combine organic and inorganic components [15,87]. These materials have been used as a suitable coating for food analysis. Peng et al. [88] developed an amide-functionalized polysaccharide–silica hybrid monolith for IT-SPME extraction of ractopamine from pork muscle. The monolith was formed from agarose oxide and tetramethoxysilane, then functionalized with amide groups through a ring-opening thiol-ene click reaction followed by dehydration. The resulting structure showed interconnected macropores and a rough skeleton with feature sizes of about 10–30 μm, enabling efficient extraction of ractopamine from complex food matrices.

Ongoing advances in IT-SPME coatings have enabled the introduction of numerous new materials. Table 1 summarizes key advantages and drawbacks of the sorbents discussed in this section and identifies the main challenges for their use as IT-SPME coatings in food matrices. Beyond the technical issues inherent to each material, food samples pose additional hurdles and often require preliminary sample preparation steps before analysis, which helps explain the limited number of studies that apply novel IT-SPME sorbents to food matrices.

A comparative analysis of sorbent materials highlights that the optimal selection inherently involves trade-offs among selectivity, robustness, and extraction efficiency. Sorbents with high surface area, such as carbon-based materials, can enhance sensitivity, especially in aqueous matrices; however, their strong intermolecular interactions often promote agglomeration, which negatively affects column reproducibility and long-term reusability. Highly selective phases are particularly advantageous for targeted analysis in lipid-rich food matrices, yet they may limit multiresidue capabilities, which are essential for routine food monitoring. Therefore, the design of new IT-SPME materials should balance analytical performance with practical implementability to ensure robustness and applicability in real food analysis.

Table 1 also provides valuable information on typical analytes and representative food matrices, while presenting key analytical performance indicators for the coating materials. It is necessary to clarify that the information presented in Table 1 should be regarded only as a proposed classification within the scope of this study and does not represent a universal statement. Typical applications associated with specific analytes or food matrices cannot be generalized, since the optimization process largely governs the extraction behavior of each sorbent. During method optimization, the performance of a given coating phase can be tuned to maximize the response to a specific class of compounds and food matrices. Nevertheless, Table 1 may still offer useful general insights into common application scenarios. For instance, RAM coatings are frequently employed in protein- and lipid-rich food matrices, such as milk and meat, owing to their inherent size-exclusion and cleanup capabilities.

In contrast, when preconcentration and selectivity are the primary objectives, more specific coating phases capable of molecular recognition, such as MIPs, may be preferred, for example, in juice samples for tracing contaminants like pesticides. Carbon-based materials also represent an attractive strategy for trace-level determinations owing to their high adsorption capacity, large surface area, and strong preconcentration potential. These considerations should be interpreted as general trends rather than definitive rules. Once again, the application of any coating phase must be optimized according to the target compounds and food matrices, particularly during the IT-SPME method optimization step, regardless of the sorbent material employed.

It is important to note that Section 3 of this review presents materials that remain largely unexplored as coating strategies for IT-SPME. Their application in IT-SPME remains limited, and because this review focuses on food analysis, the studies discussed are limited to that context. Our critical appraisal reveals a clear gap in the adoption of novel materials for IT-SPME in food analysis. This gap is evident not only in the small number of available studies but also in their recency, with very few publications in the past two years addressing these materials in food-related IT-SPME applications. This is also a call for researchers to recognize the potential of IT-SPME not only as a powerful tool for biological analysis but also as an integrated online strategy for food monitoring, using coating materials that are not yet described in the literature. Materials that perform well in biological or environmental samples often exhibit limited transferability to food matrices due to their greater complexity (e.g., lipids, carbohydrates, and particulate matter), and alternative strategies are required. With focused effort, this approach can open new horizons for food monitoring and for chemical profile evaluation, delivering meaningful advances for the broader analytical science community. The applicability of IT-SPME coatings depends on their compatibility with the chromatographic system, which is fundamental to the implementation of novel materials in robust, automated analytical workflows.

## 4. Coupling with Liquid Chromatography

The integration of IT-SPME with LC is a key feature of this technique, as it enables automated sample preparation while reducing solvent consumption and minimizing manual handling by combining extraction, desorption, and separation in a single workflow [20]. This coupling can be implemented using different configurations, such as draw–eject and flow-through systems, which rely on valves and connectors to interface the capillary device with the chromatographic system [89]. However, some challenges persist, including clogging (particularly in packed capillaries) and the need for compatibility between the extraction phase and the mobile-phase solvents to ensure efficient desorption of the target analytes. Although IT-SPME has traditionally been coupled with LC methods [90,91], recent developments have expanded its application to advanced chromatographic platforms, including UPLC, CapLC, and NanoLC [15]. This section provides a detailed overview of the coupling systems and chromatographic configurations employed.

### 4.1. Draw-Eject and Flow-Through Systems

**Figure 5:**
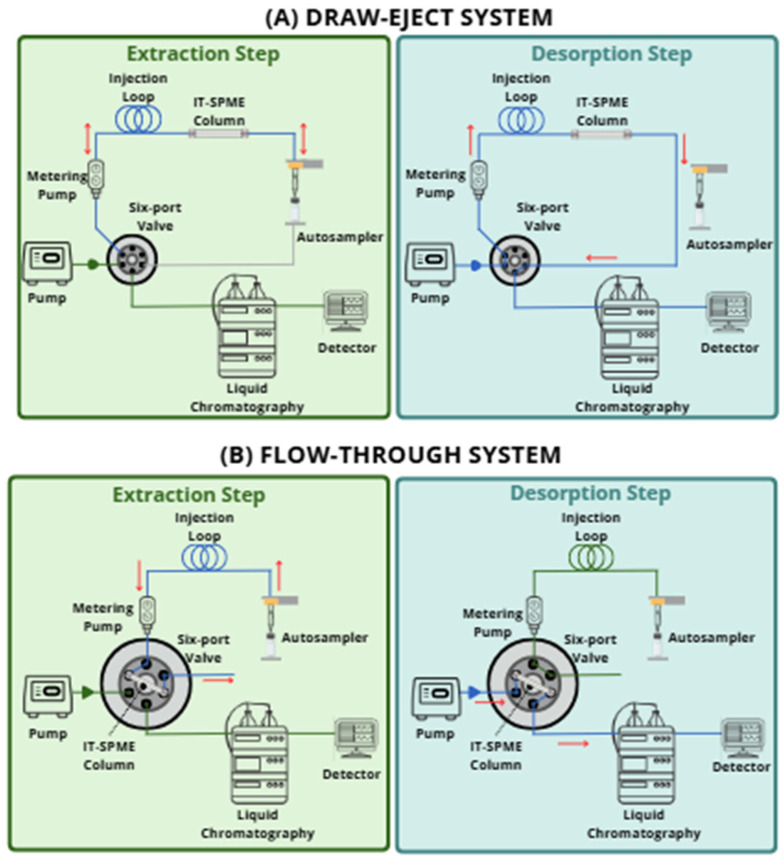
Schematic illustration of (**A**) draw-eject and (**B**) flow-through systems. Adapted from Kataoka [15].

In IT-SPME, operations such as extraction, concentration, desorption, and injection can be fully automated using column-switching technology integrated with programmable autosamplers [15]. IT-SPME can be classified into two main configurations: the draw-eject extraction system and the flow-through extraction system [25]. Figure 5 illustrates these configurations.

In the draw-eject mode (Figure 5A), the first IT-SPME system ever proposed, a capillary column is connected between the injection loop and the LC autosampler needle. In this system, target analytes are extracted by repeatedly suctioning and ejecting the sample solution through the capillary column until near-equilibrium sorption is reached, typically after 25–30 cycles [23]. Because the solution movement occurs exclusively during these cycles, contamination of the dosing pump and the switching valve is avoided. Precise control over flow rate and cycle number is generally achieved using a programmable autosampler, although manual operation is also possible in non-automated systems [25,92].

As shown in Figure 5A, the system controls the injection syringe, which repeatedly aspirates and ejects the sample from the vial, promoting the partitioning of analytes from the matrix into the stationary phase. Subsequently, the extracted analytes can be desorbed directly from the capillary coating by the flow of the mobile phase or by a desorption solvent aspirated after switching the six-port valve. The desorbed analytes are then transferred to the LC column, where they are separated and detected using an appropriate detector, like an ultraviolet-visible detector (UV-vis detector) or MS. During analysis, the chromatographic column can be re-equilibrated simultaneously with the subsequent IT-SPME extraction, since the mobile phase flows continuously through the LC column [93].

The main advantage of this system is its ability to fully automate, enabling it to control aspiration/ejection cycles, valve switching, peripheral devices (LC and MS), and analytical data processing. This reduces manual labor, increases precision, and enables automatic processing of many samples without carryover effects, since both the needle and the capillary are rinsed with an appropriate solvent before each extraction [20]. However, drawbacks include the need for a programmable autosampler with a dosing pump, which may not be available in all LC systems, and potentially longer extraction times when a high number of cycles is required to achieve optimal extraction yields. Despite this, automation allows continuous overnight operation without constant supervision [94].

In the flow-through system (Figure 5B), different configurations can be employed to continuously percolate the sample in one direction through the capillary extraction column until the analytes are sufficiently preconcentrated. Afterwards, analytes can be desorbed either statically or dynamically and transferred to the chromatographic column. In this setup, the capillary column is connected to a six-port switching valve or directly to the analytical circuit [15].

The simplest configuration, known as IT-SPME in-valve, positions the capillary extraction tube relative to the injection valve via a loop. Generally, analyte preconcentration is performed in four main stages: conditioning, extraction, washing, and desorption. Initially, the capillary is rinsed and conditioned. During sample loading, the six-port valve is set to the loading position, allowing the solution to be continuously pumped through the capillary. The sample can be delivered using a high-pressure syringe pump or a peristaltic pump, depending on the system. After loading, the capillary is rinsed to remove residual matrix components. In the desorption step, the valve is switched to the injection position, and the LC mobile phase dynamically transfers the analytes to the column [92,94].

Although the in-valve system is relatively simple to implement, direct contact between the capillary and the valve may result in sample contamination of the valve, compromising quantitative accuracy and leading to analyte overestimation. Furthermore, the loading flow rate typically prevents complete equilibrium between the sample and the extraction phase, resulting in partial analyte retention. This effect can be minimized by increasing the sample volume to the capillary’s maximum capacity, though this approach extends the preconcentration time [95].

An alternative approach has been proposed for more complex instrument setups that use two pumps and/or two six-port valves. In such systems, a transfer loop connected to an additional valve enables more precise control of the sample volume delivered to the capillary. Depending on the circuit configuration, desorption and transfer to the column may occur in two ways: (i) the loop content is transferred to the capillary, followed by valve switching and analyte desorption by the mobile phase; or (ii) analytes are desorbed into the transfer loop, and only the fraction corresponding to the loop volume is injected into the column. A distinctive feature of this configuration is the absence of a direct connection between the capillary and the chromatographic flow, which allows extraction and separation under independent conditions and prevents the capillary from being subjected to high pressures [15,20].

The main advantages of the flow-through mode include: (i) direct and continuous coupling with the chromatographic system, facilitating integration with LC or MS and enabling highly sensitive bioanalytical applications; (ii) the ability to use large sample volumes, enhancing method sensitivity; (iii) unidirectional flow, reducing carryover and cross-contamination; and (iv) rapid and simultaneous execution of multiple extractions and analyses, reducing total analysis time. Additionally, capillary conditioning and chromatographic separation can be performed independently [96]. On the other hand, some operational disadvantages must be considered: (i) longer preconcentration times when large sample volumes are used; (ii) risk of capillary clogging by suspended particles, requiring prior sample filtration; (iii) possible valve contamination when the capillary is directly connected to the six-port valve; and (iv) the need for strict control of valve switching time and flow rate to ensure reproducible results [29].

Overall, both IT-SPME coupling modes offer high automation and compatibility with chromatographic systems. The draw-eject mode provides precise control and high reproducibility, making it more suitable for quantitative applications. In contrast, the flow-through mode offers higher sensitivity and efficiency for processing large sample volumes and facilitates direct coupling with LC-MS systems [16]. The main advantages and disadvantages of the draw-eject and flow-through IT-SPME modes are summarized in Table 2.

### 4.2. External Stimuli

Another approach to improving IT-SPME performance is the use of external stimuli, such as magnetic fields, electric fields, and temperature control. Magnetic IT-SPME employs extraction phases containing immobilized magnetic nanoparticles (MNPs), in which magnetic forces are unaffected by pH and concentration and enable controlled fluid movement within the capillary columns. Differences in magnetic susceptibility between the analyte and the sample matrix allow diamagnetic forces to be exploited for trapping and concentrating analytes in regions of minimal magnetic field. The analytes are then readily desorbed by reversing the polarity of the applied magnetic field [26,97,98]. The application of magnetic fields can accelerate extraction kinetics and improve analyte retention efficiency. The use of Fe_3_O_4_ MNPs combined with 4-vinylphenylboronic acid and ethylene in a monolithic capillary column was described by Mei et al. for the extraction of heavy metal ions from fish and shrimp samples [99]. In their study, a fused-silica capillary (20 cm × 320 µm) was packed with the magnetic monolithic material and wrapped with a magnetic coil for field application. The magnetism-reinforced IT-SPME/LC-DAD method developed enabled the determination of trace levels of Co(II), Cu(II), and Hg(II), with limit of detection (LOD) and limit of quantification (LOQ) ranging from 0.69 to 4.9 µg/kg and from 2.2 to 16 µg/kg, respectively. Magnetic sorbents have been widely explored for the analysis of contaminants and food ingredients [100], and their integration with IT-SPME can provide a simple, highly efficient sample preparation strategy for food analysis.

Electrochemically controlled IT-SPME (EC-IT-SPME) employs conducting polymers as extraction phases. This modulation, of surface charge through oxidation and reduction, enables efficient extraction of polar or ionic analytes. In this configuration, polymer-coated capillaries serve as the working electrode, and alternating the applied electric field tunes the analyte–sorbent affinity, thereby facilitating both extraction and desorption steps [101]. Temperature-controlled IT-SPME (TC-IT-SPME) allows the capillary column temperature to be varied from 0 to 100 °C using thermoelectric heating and cooling. Lower extraction temperatures can increase the partition coefficient, whereas higher desorption temperatures can enhance diffusion coefficients and accelerate analyte release. The use of thermoresponsive polymers enables switching between hydrophilic and hydrophobic states, modulating analyte–sorbent interactions and improving both extraction and desorption efficiency [102]. Despite their potential, applications of EC-IT-SPME and TC-IT-SPME in food analysis have not yet been reported until the conceptualization of this review study.

### 4.3. Chromatographic Coupling and Reported IT-SPME Applications to Food Analysis

The first publication describing the coupling of IT-SPME with a liquid chromatography-ultraviolet detection (LC-UV) system [90], and this configuration remains the most common application. The in-tube solid-phase microextraction coupled to liquid chromatography (IT-SPME-LC) hyphenation provides excellent performance in terms of reproducibility, sensitivity, and rapid analyses, supporting high-throughput workflows. Table 3 summarizes selected IT-SPME applications from the last six years for the determination of analytes in various food matrices using different chromatographic detection systems.

Several publications have reported the use of online IT-SPME-LC with various detection modes. For example, Zhao et al. developed a functional affinity monolithic (Apt@EDV) column and a polymerized oligonucleotide-grafted hydrophilic affinity monolithic column for in-tube solid-phase microextraction coupled to liquid chromatography with fluorescence detection (IT-SPME-LC-FLD) [103,104]. Fused-silica capillaries of 5 cm × 75 µm i.d. (Apt@EDV) and 20 cm × 100 µm i.d. (hydrophilic monolith) were used. Both columns were applied for the selective extraction of OTA in food matrices, achieving excellent LODs. A fiber-in-tube solid-phase microextraction coupled to liquid chromatography with ultraviolet detection (IT-SPME-LC-UV) approach was described by Yuan et al. for the analysis of Sudan dyes in chili [108]. Stainless steel fibers were modified with NiAl-layered double hydroxides and packed into PEEK tubing with 0.02 in i.d. Parameters such as sample volume, sampling rate, sample pH, and acetonitrile content in the sample solution were optimized, and the method showed good reproducibility and high sensitivity, with LODs ranging from 0.1 to 0.2 ng/mL.

Sun et al. reported a covalent organic framework combined with carbonized carbon fibers (CFs@C-COF), characterized by a high surface area, abundant mesopores, and excellent durability [38]. The IT-SPME device was fabricated from 20 cm PEEK tubing with a 1 mm i.d., and coupling to liquid chromatography with diode-array detection (LC-DAD) was performed in a flow-through configuration. Factors such as sample solution volume, sampling rate, and desorption time were optimized, and the extraction of seven PAEs from Chinese Baijiu yielded LODs and LOQs of 0.3–0.5 µg/L and 1.0–1.5 µg/L, respectively. A stainless-steel IT-SPME device (10 mm × 3 mm i.d.) containing a monolithic material based on polyhedral oligomeric silsesquioxane (POSS) was proposed by Liu et al. for the determination of bisphenols (BPs) in milk [114]. The polymer was synthesized via thermal polymerization using azobisisobutyronitrile (AIBN) as the initiator, and the combination of POSS with acrylamide (AM) co-polymerized with ethylene dimethacrylate (EDMA) yielded an extraction phase with excellent mechanical stability and high sensitivity, achieving LODs of 0.030–0.055 ng/mL. The online IT-SPME–LC–UV system was operated using a six-port valve that alternated between the “LOAD” and “INJECT” positions.

Another noteworthy monolithic material was developed by Wang et al. for the analysis of edible oils [115]. The doping of covalent organic framework (COF) microspheres with urea-formaldehyde (UF) resins, which are highly hydrophilic, enabled the preparation of 20 cm PTFE capillary columns (750 µm i.d.) with excellent extraction efficiency for SPAs, achieving LODs of 0.2–1.2 ng/mL. The online IT-SPME–LC–UV coupling was evaluated by varying sample and eluent composition, pH, desorption time, and flow rates. Additionally, Feng et al. reported a 30 cm PEEK extraction tube (75 µm i.d.) packed with stainless-steel wires coated with dendritic mesoporous silica nanospheres bearing porous carbon on their surface. After optimization, the in-tube solid-phase microextraction coupled to liquid chromatography with diode array detection (IT-SPME–LC–DAD) method enabled the extraction of PAHs from tea samples, yielding LODs of 0.010–0.070 µg/L and demonstrating the broad applicability of carbon-based materials.[117]

IT-SPME can also be applied for off-line sample preparation. In this approach, the sample solution is passed through the extraction capillary, where the analytes are retained and subsequently desorbed. The resulting extract is then injected into the chromatographic system for separation and detection, allowing additional clean-up or derivatization steps to improve compatibility with the analytical platform. Another advantage of off-line IT-SPME is the ability to use simpler instrumental configurations, since the extraction column does not need to be directly coupled to the chromatographic system.

Chen et al. reported a magnetism-assisted in-tube solid-phase microextraction (MA-IT-SPME) approach employing a monolithic capillary column (MCC) [110]. A fused-silica capillary with a 320 µm i.d. was used, and the Fe_3_O_4_-doped monolithic phase was synthesized in situ via thermal initiation polymerization. A magnetic coil was wrapped around the capillary column, enabling the direction and magnitude of the magnetic field to be adjusted. For the extraction of phenolic acids from juice samples, a 20 Gs magnetic field was applied in the same direction as the sample solution flow. For desorption, a 40 Gs magnetic field with reversed polarity was employed. The extracted analytes were subsequently analyzed off-line by LC–DAD.

An interesting analysis of functional foods was reported by Dong et al., in which PDE-5 inhibitors were extracted using IT-SPME and analyzed off-line by LC–UV [111]. Thermal polymerization yielded a poly(VBIMBr–EDMA) monolithic column prepared in a 10 cm fused-silica capillary with a 250 µm i.d., and its extraction performance was evaluated by optimizing extraction parameters, including sample solvent, elution solvent, flow rates, and loading volumes of both sample and elution solvent. The method exhibited excellent reproducibility, achieving LODs of 0.5–0.9 ng/mL. Nasrollahi et al. developed electrospun nanofibers based on chitosan and poly(vinyl alcohol) (CS@PVA) for the determination of acidic red dyes in juice samples [75]. The nanofibers were packed into stainless-steel tubes (50 mm × 1.8 mm i.d.), and extraction was carried out using a peristaltic pump. Desorption was performed using a syringe pump, and after completion, the analytes were separated and detected by LC–UV.

Another interesting approach employing IT-SPME for sample preparation was described by Aghaziarati et al. [112]. Carbon dot–amino acid hybrids intercalated into layered double hydroxides (LDHs) were used as the extraction phase, packed into 100 mm-long, 0.8 mm i.d. copper tubes, for the extraction of chlorophenols from juice and honey samples. The extraction of benzodiazepines from juice samples was investigated by Tahmasebi and Aghajanzadeh [113]. In their study, a 1 cm stainless-steel column (0.4 cm i.d.) was packed with an oxygen- and nitrogen-doped mesoporous graphitic carbon–iron carbide nanocomposite (MGC/IC NC), and the sample solution was circulated through the tube using a peristaltic pump for 25 min. The eluate was subsequently analyzed by LC–UV, and the nanocomposite exhibited remarkable stability and high extraction efficiency, achieving LODs of 0.02–0.20 µg/L. Saadat et al. synthesized magnetic polymer composite particles (MPCPs) consisting of an Fe_3_O_4_ magnetic core modified with oleic acid (OA) and a divinylbenzene (DVB) polymer shell, which were employed for the off-line extraction of triazines from orange, cucumber, and onion samples [109].

The coupling of IT-SPME with LC–MS strategies is also well established. MS detectors offer higher sensitivity and specificity, thereby improving analyte identification and quantification and expanding the range of target compounds that can be monitored. Ishizaki et al. investigated the determination of luteolin and apigenin in herbal teas using an in-tube solid-phase microextraction coupled to liquid chromatography–tandem mass spectrometry (IT-SPME–LC–MS/MS) strategy [32]. In their method, a Supel Q PLOT column was employed in a modified draw-eject system. The column was connected between the autosampler and a six-port valve. With the valve in the extraction position, the sample solution was continuously aspirated and ejected through the column; thereafter, the analytes were desorbed by switching the valve to the desorption position. The resulting fully automated online analytical method provided high sensitivity and selectivity while remaining environmentally friendly. An in-tube solid-phase microextraction coupled to hydrophilic interaction liquid chromatography–tandem mass spectrometry (IT-SPME–HILIC–MS/MS) approach was described by Zhang et al. [41]. Halloysite nanotubes (HNTs), modified with amine–PEG–vinyl, acrylamide (AM), and N,N’-methylenebisacrylamide (MBA), were packed into polyimide-coated fused-silica capillaries (10 cm × 530 µm i.d.). After optimization of the IT-SPME procedure, cationic pesticides (maleic hydrazide, amitrole, and cyromazine) were quantified in cucumber, carrot, apple, kidney bean, and eggplant samples, achieving LODs ranging from 0.1 to 2.1 µg/kg. The newly synthesized extraction phase exhibited excellent permeability, good reusability and reproducibility, and outstanding adsorption capacity.

Coupling IT-SPME to UPLC systems may pose challenges, as the sample-preparation device operates at low pressure, whereas the chromatographic system operates at high pressure. However, this issue can be easily addressed by incorporating six-port valves. An in-tube solid-phase microextraction coupled to ultra-performance liquid chromatography–tandem mass spectrometry (IT-SPME–UPLC–MS/MS) approach was described by Zhang et al. [106]. For this purpose, fused-silica capillaries (10 cm × 530 µm i.d.) were packed with a hybrid MOF–polymer monolithic material (NH_2_-MIL-53(Al)) and connected to a six-port valve for the extraction of sulfonamides (SAs) in chicken and fish samples. Good selectivity and high recoveries were achieved, with an LOD value of 1.3–4.7 ng/L.

In 2020, Carmona and Lanças reported the coupling of IT-SPME–UPLC–MS/MS in the flow-through extraction mode, using a Supelco six-port external valve [49]. The sorbent employed was SiGOC18ecap packed in a stainless-steel capillary tube (100 mm × 0.5 mm i.d.), and parameters such as loading time, injection volume, loading flow rate, and solvent type were evaluated. The optimized sample-preparation procedure was applied to the extraction of xanthines from coffee samples and proved suitable, rapid, and straightforward, with LODs of 0.10–0.20 µg/L. A fully automated approach for mycotoxin analysis based on IT-SPME–UPLC–MS/MS was described by Maciel et al. [105]. In that study, two stainless-steel capillary columns were investigated, packed with SiGOC18ecap (100 mm × 508 µm i.d.) and GOSil (100 mm × 254 µm i.d.). A six-port valve was used for conditioning, elution, and column clean-up steps. Wine, coffee, and almond liquor samples were analyzed, and the proposed method for mycotoxin determination exhibits excellent performance, benefiting from miniaturization and automation. Miniaturized LC systems, such as CapLC and nanoLC, can also be coupled to IT-SPME. IT-SPME–CapLC coupling can be achieved with minimal modifications. For coupling with nanoLC, however, the dimensions of the IT-SPME capillary column must be compatible and adjusted with the very low flow rates employed. These systems offer several advantages, including high sensitivity, straightforward hyphenation with MS detectors, and significant reductions in solvent consumption. Although this coupling strategy is well established, it is predominantly applied to biological and environmental matrices, with only a few reports describing applications in food analysis [118].

Finally, IT-SPME can also be coupled to other analytical techniques, such as capillary electrophoresis (CE), inductively coupled plasma mass spectrometry (ICP–MS), atomic absorption spectrometry (AAS), capillary electrochromatography (CEC), and direct MS. In offline configurations, it can also be hyphenated to GC and matrix-assisted laser desorption/ionization-time-of-flight (MALDI–TOF–MS) [15]. The coupling between IT-SPME and CEC was described by Lei et al. [116]. Fused-silica capillaries (5 cm × 530 µm i.d.) were filled with a poly(MPC-co-ILs-co-TMA) monolith. The procedure was performed using glass syringes, and the extraction phase exhibited good extraction efficiency, low LODs (5.0–10.0 µg/L), and short extraction times for glycopeptide antibiotics in pork samples. Wu et al. reported an IT-SPME–direct MS coupling approach [107]. A 20 cm fused-silica column (200 µm i.d.) packed with poly(AAPBA-co-DVB-co-MBAA), and two pumps were connected to the system. In this system, pump A delivered the sample solution (extraction), while pump B provided the desorption solvent. After optimization of the IT-SPME conditions, the method was applied to the extraction and detection of benzimidazole in chicken and pork, showing good extraction efficiency and precision, achieving LODs of 0.55–0.91 ng/g.

The derivatization process can also be implemented online in IT-SPME [119]. Derivatization can be performed post-column (PCD), or the reagent can be added directly into the capillary, where the passage of the sample solution simultaneously extracts and derivatizes the analytes, which are then desorbed into the analytical column. Automating this step reduces sample handling, reduces reagent consumption, and minimizes waste generation, making it highly attractive from a green sample-preparation perspective. However, no applications of this approach for food analysis have yet been reported.

Despite the potential of IT-SPME for food analysis, there is an apparent lack of applications in this field relative to its widespread use in biological studies. In the author’s view, this limited, outdated literature underscores the need for researchers to develop new IT-SPME protocols tailored to specific food matrices. This gap is also evident in the development and application of novel sorbent coatings for IT-SPME. Lab-made materials represent a promising alternative that can expand the range of applications to complex food matrices while offering new opportunities to replace traditional, costly commercial IT-SPME columns. Recent advances in food analysis indicate that IT-SPME strategies may soon reach a new stage of automation, enabling the routine evaluation of various compounds, including contaminants, with relevance for food safety.

## 5. Conclusions and Future Trends

As demonstrated in this review, IT-SPME is an emerging technique for developing miniaturized, modern, and automated sample preparation protocols. By combining cleanup, extraction, and preconcentration into a single step, this approach provides an environmentally friendly, efficient extraction method. The fabricated columns vary in length and inner diameter, and these parameters must be optimized based on the specific analytical system. New hybrid materials with unique characteristics have been developed for use in this technique, and their integration with fully automated systems demonstrates the robustness of the methods. IT-SPME is highly versatile, pairing well with advanced analytical techniques, integrating with UPLC and miniaturized LC systems, and connecting directly to MS, highlighting the technique’s potential.

Regarding future trends and directions for IT-SPME in food analysis, two main horizons can be identified. The first concerns the development of novel coating materials, and the second involves the integration of IT-SPME with miniaturized LC systems. Advanced sorbent materials such as MOFs, COFs, MIPs, magnetic nanoparticles, and monolithic phases, together with sustainable bio-based coatings incorporating aptamers, antibodies, and natural polymers, have the potential to overcome the limitations of conventional coatings. In addition to offering versatility across physicochemical properties, many of these materials enable greener strategies for coating phases, aligning with Green Analytical Chemistry principles.

The integration of IT-SPME with miniaturized LC systems and the use of external stimuli, such as magnetic or electric fields and temperature, may further expand its analytical capabilities. Coupling IT-SPME with CapLC and NanoLC can enhance analytical performance in specific applications and represents an essential direction toward greener methodologies, due to reduced solvent and sample consumption. Moreover, coupling with direct MS and other advanced detection systems can broaden the scope of the technique. The direct introduction of extracted analytes into the MS system minimizes sample handling, decreases contamination risks, and improves reproducibility. The high enrichment capacity of IT-SPME also increases the amount of analyte reaching the ion source, thereby enhancing detection limits for trace and ultra-trace compounds. Despite these advances, significant challenges remain in applying IT-SPME to food matrices, as the complex, heterogeneous composition of foods demands robust, adaptable analytical strategies. Even so, ongoing developments indicate that IT-SPME may soon enter a new phase of enhanced automated workflows, offering powerful tools for food science, including contaminant monitoring and the chemical profiling of challenging matrices.

Overall, this review highlights the need to advance IT-SPME in food analysis by integrating novel sorbents, device configurations, and chromatographic compatibility. Future research on IT-SPME applied to food analysis should focus on current analytical demands, such as regulatory monitoring and routine control. The implementation of high-throughput, multiresidue workflows, as well as strategies to minimize the effects of complex food matrices, is essential for developing robust methods capable of detecting contaminants at trace levels. These efforts may broaden the applicability of IT-SPME in food science and contribute to the establishment of more efficient, sustainable, and reliable analytical approaches for food safety assessment.

## Figures and Tables

**Table 1:** Overview of IT-SPME coating materials for food analysis: analyte classes, matrix suitability, and performance considerations.

Coating Material	Typical Analyte Classes	Food Matrix Types	Key Analytical Performance Indicators	Advantages	Drawbacks	Challenges for IT-SPME in Food Samples
Carbon-based materials (graphene, CNTs, CNFs, fullerenes, etc.)	Non-polar and semi-volatile compounds, PAHs, pesticides, flavor compounds, endocrine disruptors	Aqueous food extracts, beverages, low-fat foods, diluted food matrices	High surface area, strong π–π interactions, high enrichment factors, good thermal stability, moderate selectivity	Large surface area; strong π–π and hydrophobic interactions; tunable surface chemistry; good thermal and mechanical stability when hybridized; reusability	Agglomeration and fines migration; backpressure increase; poor packing reproducibility; mechanical fragility; batch variability	Controlling uniform packing; avoiding clogging; improving adhesion inside capillaries; extending applicability to complex food matrices
Molecularly imprinted polymers (MIPs)	Target-specific analytes such as antibiotics, parabens, hormones, veterinary drugs	Complex food matrices including milk, meat products, cereals, and beverages	High selectivity, good repeatability, low matrix interference, moderate extraction capacity	High selectivity due to molecular recognition; good chemical and thermal stability; reusable	Template bleeding; heterogeneous binding sites; mechanical brittleness; difficult immobilization	Complete template removal; minimizing backpressure; reducing site heterogeneity; reproducible capillary integration
Restricted access materials (RAM)	Small molecules in the presence of proteins and lipids such as drugs, additives, pesticides	Protein- and lipid-rich foods such as milk, meat, dairy products, eggs	Reduced matrix effects, selective macromolecule exclusion, good robustness, compatibility with LC	Selective exclusion of macromolecules; reduced matrix effects; good reusability; LC compatibility	Complex synthesis; instability under extreme pH or solvents; variable retention	Controlling pore size and diffusion barriers; avoiding backpressure; ensuring stability in diverse food matrices
Bio-based materials (chitosan, cellulose, cyclodextrin, etc.)	Polar and moderately polar compounds, phenolics, mycotoxins, additives	Plant-based foods, fruits, vegetables, beverages, moderately complex matrices	Green character, functional group availability, moderate selectivity, limited long-term robustness	Renewable and environmentally friendly; rich functional groups; low toxicity; hybridization potential	Chemical instability; mechanical fragility; packing variability; surface fouling	Improving mechanical robustness; uniform functionalization; preventing fouling; reproducibility across natural sources
Ionic liquids (ILs)	Polar to semi-polar analytes, pharmaceuticals, preservatives, flavor compounds	Liquid foods, beverages, aqueous extracts	Tunable polarity, good extraction efficiency, low vapor pressure, moderate stability	Tunable properties; negligible vapor pressure; high thermal stability; reusable	High cost; synthesis complexity; possible leaching; limited food applications	Stable immobilization; compatibility with complex matrices; long-term coating stability
Magnetic nanomaterials (MNMs)	Diverse organic contaminants including pesticides, drugs, and additives	Liquid and semi-liquid food matrices, viscous extracts	High surface area, fast kinetics, enhanced desorption efficiency, magnetic control	Superparamagnetic behavior; easy manipulation; enhanced extraction efficiency	Aggregation; oxidation; magnetic field dependence; synthesis complexity	Reproducible magnetic integration; stable magnetization; efficient desorption in viscous matrices
Aerogels	Volatile and semi-volatile organic compounds, hydrophobic analytes	Mainly aqueous food samples and beverages	Extremely high porosity, high adsorption capacity, low density	High porosity; tunable pore structure; excellent adsorption; reusable	Fragility; complex synthesis; limited food applications	Application to complex matrices; improving mechanical strength; synthesis standardization
Monolithic capillaries	Wide range of analytes including pesticides, drugs, and additives	Complex and viscous food matrices, extracts with suspended solids	Low backpressure, high permeability, fast mass transfer, good reproducibility	Continuous porous structure; low backpressure; fast mass transfer; reproducible fabrication	Limited surface modification; shrinkage or cracking	Expanding functionalization options; multi-analyte food applications; robustness under viscosity variations

**Abbreviations: CNTs:** carbon nanotubes; **CNFs:** carbon nanofibers; **MIPs:** molecularly imprinted polymers; **RAM:** restricted access materials; **ILs:** ionic liquids; **MNMs:** magnetic nanomaterials.

**Table 2:** Key differences between draw–eject and flow-through operational modes in IT-SPME, with emphasis on extraction, transfer to column, advantages, and disadvantages.

Characteristic	Draw-Eject Mode	Flow-Through Mode
Extraction	Partial; equilibrium is rarely achieved.	Higher preconcentration efficiency; Adjustable by sample volume
Transfer to column	Via repeated ejection; less direct.	Dynamic transfer through mobile phase; May be static or via transfer loop.
Advantages	Simple implementation; Low clogging risk; Requires smaller sample volumes.	Direct coupling to the LC system; Allows large sample volumes; Unidirectional flow minimizes carryover; Extraction and separation can occur under different conditions.
Disadvantages	Incomplete extraction; Limited sensitivity; Requires multiple cycles and longer times.	Longer preconcentration time for large volumes; capillary clogging risk; Possible valve contamination; Requires strict control of flow rate and valve switching.

**Table 3:** Summary of the applications of IT-SPME in food matrices over the last six years.

Matrix	Analyte	Extraction Phase	Column Material	Column Dimensions	Separation/Detection System	LOD	Ref.
Beer and wheat	OTA	Apt@EDV	Fused silica	5 cm × 75 µm i.d.	LC-FLD	0.025 ng/mL	[103]
Beer and wine	OTA	oligonucleotideo-grafted hydrophilic affinity monolith	Fused silica	20 cm × 100 µm i.d.	LC-FLD	0.012 ng/mL	[104]
Beverages	OTA	SiGOC18ecap	Stainless-steel	100 mm × 508 µm i.d.	UPLC-MS/MS	68–351 ng/L*	[105]
	OTA, ZEA, and AFAs	GOSil	Stainless-steel	100 mm × 254 µm i.d.	UPLC-MS/MS	110–1110 ng/L*	
Chicken and fish	Sulfonamides	NH_2_-MIL-53(Al)	Fused silica	15 cm × 530 µm i.d.	UPLC-MS/MS	1.3–4.7 ng/L	[106]
Chicken and pork	Benzylimidazole	poly(AAPBA-co-DVB-co-MBAA	Fused silica	20 cm × 200 µm i.d.	MS	0.55–0.91 ng/g	[107]
Chili	Sudan dyes	NiAl-LDHs	PEEK	0.02 in. i.d.	LC-UV	0.1–0.2 ng/mL	[108]
Chinese Baijiu	PAEs	CFs@C-COF	PEEK	20 cm × 1 mm i.d.	LC-DAD	0.3–0.5 µg/L	[38]
Coffee	Xanthines	SiGOC18ecap	Stainless-steel	100 mm × 0.5 mm i.d.	UPLC-MS/MS	0.10–0.20 μg/L	[49]
Fruit and vegetables	Triazines	Fe_3_O_4_@PS/DVB-MNPs	PP	2 cm × 1.5 mm i.d.	LC-UV	0.1 µg/L	[109]
Fruits and vegetables	Pesticides	Halloysite nanotubes	Fused silica	10 cm × 530 µm i.d.	HILIC-MS/MS	0.1–2.1 µg/kg	[41]
Fruit juices	Phenolic acids	MCC@Fe3O4	Fused silica	320 µm i.d.	LC-DAD	0.012–0.061 µg/L	[110]
Functional foods	PDE-5 inhibitors	Poly (VBIMBr-EDMA)	Fused silica	10 cm × 250 µm i.d.	LC-UV	0.5–0.9 ng/mL	[111]
Herbal teas	Luteolin e apigenin	Supel Q PLOT	Fused silica	60 cm × 0,32 mm i.d.	LC-MS/MS	0.4–0.8 pg/mL	[32]
Honey, juice	Chlorophenols	C-dots@His/LDHs	Copper	100 mm × 0.8 mm i.d.	LC-UV	0.1–1.0 μg/L	[112]
Juices	Acidic red dyes	CS@PVA nanofibers	Stainless-steel	50 mm × 1,8 mm i.d.	LC-UV	0.3–7.6 ug/L	[75]
Juices	Benzodiazepines	MGC/IC NC	Steel	1 cm × 0.4 cm i.d.	LC-UV	0.02–0.20 µg/L	[113]
Milk	Bisphenols	POSS-co-AM-co-EDMA	Stainless-steel	10 mm × 3 mm i.d.	LC-UV	0.030–0.055 ng/mL	[114]
Oil	SPAs	COFTAPB-TPA@UF	PTFE	20 cm × 750 µm i.d.	LC-UV	0.2–1.2 ng/mL	[115]
Pork	Glycopeptide antibiotics	Poly (MPC-co-ILs-co-TMA)	Fused silica	5 cm × 530 µm i.d.	LC-MS	5.0–10.0 µg/L	[116]
Tea	PAHs	DMSNs@porous carbon	PEEK	30 cm × 0.75 mm i.d.	LC-DAD	0.010–0.070 µg/L	[117]

**Abbreviations: AFAs:** Aflatoxins. **Apt@EDV:** Aptamer@Poly(Ethylene Dimethacrylate-co-methacrylatoethyltrimethyl ammonium chloride-co-2-vinyl-4,4-dimethylazlactone). **C-dots@His/LDHs:** Carbon-dots@Histidine/layered double hydroxides. **CFs@C-COF:** Carbon Fibers@Carbonized-Covalent Organic Frameworks. **COFTAPB-TPA@UF:** Covalent Organic Framework-1,3,5-Tris (4-aminophenyl) benzene-terephthaldicarldehyde@Urea-formaldehyde. **CS@PVA nanofibers:** Chitosan@polyvinyl alcohol nanofibers. **DMSNs@porous carbon:** Dendritic mesoporous silica nanospheres@porous carbon. **Fe_3_O_4_@PS/DVB-MNPs:** Fe_3_O_4_@persulfate/divinylbenzene-magnetic nanoparticles. **GOSil:** Graphene Oxide anchored on aminopropyl silica. **MCC@Fe_3_O_4_:** monolithic capillary column@Fe_3_O_4_. **MGC/IC NC:** mesoporous oxygen and nitrogen-doped graphitic carbon/iron carbide nanocomposite. **NH_2_-MIL-53(Al):** NH_2_-Materials of Institute Lavoisier-53(Al). **NiAl-LDHs:** NiAl-Layered Double Hydroxides. **OTA:** Ochratoxin A. **PAEs:** Phthalates. **PAHs:** Polycyclic Aromatic Hydrocarbons. **PDE-5:** Phosphodiesterase-5. **PEEK:** Polyetheretherketone. **Poly (MPC-co-ILs-co-TMA):** Poly (2-methacryloyloxyethyl phosphorylcholine-co-ionic liquids-co-trimethylolpropane trimethacrylate). **Poly (VBIMBr-EDMA):** Poly (1-butyl-3-vinylimidazolium bromide- ethylene glycol dimethacrylate. **Poly(AAPBA-co-DVB-co-MBAA):** poly (3-acrylamidophenylboronic acid-co-divinylbenzene-co-N′-methylenediacrylamide). **POSS-co-AM-co-EDMA:** Polyhedral oligomeric silsesquioxane-co-Acrylamide-co-ethylene dimethacrylate. **PP:** polypropylene. **PTFE:** Polytetrafluoroethylene. **SiGOC18ecap:** Graphene Oxide anchored on aminopropyl silica functionalized with octadecylsilane and trimethylsilane. **SPAs:** synthetic phenolic antioxidants. **ZEA:** Zearalenone. *LOQ instead of LOD.

## Data Availability

This article is a review of previously published studies. All data supporting the conclusions of this review are available within the cited references.

## Abbreviations

The following abbreviations are used in this manuscript:

AAS	atomic absorption spectrometry
AAPBA	3-acrylamidophenylboronic acid
AFAs	aflatoxins
AIBN	azobisisobutyronitrile
AM	acrylamide
CapLC	capillary liquid chromatography
C-DOTS	carbon-dots
CEC	capillary electrochromatography
CE	capillary electrophoresis
CFs	carbon fibers
CNTs	carbon nanotubes
COF	covalent organic framework
CS	chitosan
DAD	diode array detection
DESs	deep eutectic solvents
DMSNs	dendritic mesoporous silica nanospheres
DVB	divinylbenzene
EC-IT-SPME	electrochemically controlled in-tube solid phase microextraction
EDMA	ethylene dimethacrylate
FLD	fluorescence detection
GC	gas chromatography
G	graphene
GO	graphene oxide
GOSil	graphene oxide anchored on aminopropyl silica
HNTs	halloysite nanotubes
His	histidine
IC	iron carbide
ICP-MS	inductively coupled plasma mass spectrometry
ILs	ionic liquids
IT-SPME	in-tube solid phase microextraction
LC	liquid chromatography
LDHs	layered double hydroxides
LOD	limit of detection
LOQ	limit of quantification
MA-IT-SPME	magnetism-assisted in-tube solid phase microextraction
MALDI–TOF–MS	matrix-assisted laser desorption/ionization-time of flight mass spectrometry
MBAA	methylenediacrylamide
MCC	monolithic capillary column
MGC	mesoporous graphitic carbon
MIPs	Materials of Institute Lavoisier-53
MIL	molecularly imprinted polymers
MNMs	magnetic nanomaterials
MNPs	magnetic nanoparticles
MOFs	metal–organic frameworks
MS	mass spectrometry
MSPE	magnetic solid phase extraction
MWNTs	multi-walled carbon nanotubes
NanoLC	nano liquid chromatography
NC	nanocomposite
NIP	non-imprinted polymer
OTA	ochratoxin A
PAEs	phthalates
PAHs	polycyclic aromatic hydrocarbons
PDE-5	phosphodiesterase-5
PCD	post-column derivatization
PDMS	polydimethylsiloxane
PEEK	polyetheretherketone
PLOT	porous-layer open tubular
PP	polypropylene
PPY	polypyrrole
POSS	polyhedral oligomeric silsesquioxane
PS	persulfate
PTFE	polytetrafluoroethylene
PVA	polyvinyl alcohol
RAM	restricted-access material
RAMIP	restricted access–molecularly imprinted polymer
rGO	reduced graphene oxide
SAs	sulfonamides
SPE	solid phase extraction
SPAs	synthetic phenolic antioxidants
SPME	solid phase microextraction
SWNTs	single-walled nanotubes
TAPB	1,3,5-Tris(4-aminophenyl)benzene
TC-IT-SPME	temperature-controlled in-tube solid phase microextraction
TMA	trimethylolpropane trimethacrylate
TPA	terephthaldicarldehyde
UF	urea–formaldehyde
UPLC	ultra performance liquid chromatography
UV	ultraviolet
VBIMBr	1-butyl-3-vinylimidazolium bromide
WCOT	wall-coated open tubular
ZEA	Zearalenone
